# Patient-derived tumor organoids as a platform of precision treatment for malignant brain tumors

**DOI:** 10.1038/s41598-022-20487-y

**Published:** 2022-09-30

**Authors:** Chun-Chung Chen, Hong-Wei Li, Yuan-Liang Wang, Chuan-Chun Lee, Yi-Chun Shen, Ching-Yun Hsieh, Hung-Lin Lin, Xian-Xiu Chen, Der-Yang Cho, Ching-Liang Hsieh, Jeng-Hung Guo, Sung-Tai Wei, John Wang, Shao-Chun Wang

**Affiliations:** 1 Graduate Institute of Integrated Medicine, China Medical University, Taichung, Taiwan, ROC; 2 School of Medicine, China Medical University, Taichung, Taiwan, ROC; 3 Department of Neurosurgery, China Medical University Hospital, 2 Hsueh-Shih Road, Taichung City, 40402 Taiwan, ROC; 4 Graduate Institute of Biomedical Sciences, College of Medicine, China Medical University, Taichung, 40402 Taiwan, ROC; 5 Center for Molecular Medicine, China Medical University Hospital, Taichung, 404332 Taiwan, ROC; 6 Research Center for Cancer Biology, China Medical University, Taichung, 40402 Taiwan, ROC; 7 Department of Internal Medicine, China Medical University Hospital, Taichung, Taiwan, ROC; 8 Chinese Medicine Research Center, China Medical University, Taichung, Taiwan, ROC; 9 Graduate Institute of Acupuncture Science, China Medical University, Taichung, Taiwan, ROC; 10 Department of Pathology, China Medical University Hospital, Taichung, 40447 Taiwan, ROC; 11 Department of Biotechnology, Asia University, Taichung, 41354 Taiwan, ROC; 12 Department of Cancer Biology, University of Cincinnati, Cincinnati, OH 45267 USA

**Keywords:** Cancer, Cell biology, Neuroscience

## Abstract

Malignant brain tumors consist of malignancies originated primarily within the brain and the metastatic lesions disseminated from other organs. In spite of intensive studies, malignant brain tumors remain to be a medical challenge. Patient-derived organoid (PDO) can recapitulate the biological features of the primary tumor it was derived from and has emerged as a promising drug-screening model for precision therapy. Here we show a proof-of-concept based on early clinical study entailing the organoids derived from the surgically resected tumors of 26 patients with advanced malignant brain tumors enrolled during December 2020 to October 2021. The tumors included nine glioma patients, one malignant meningioma, one primary lymphoma patient, and 15 brain metastases. The primary tumor sites of the metastases included five from the lungs, three from the breasts, two from the ovaries, two from the colon, one from the testis, one of melanoma origin, and one of chondrosarcoma. Out of the 26 tissues, 13 (50%) organoids were successfully generated with a culture time of about 2 weeks. Among these patients, three were further pursued to have the organoids derived from their tumor tissues tested for the sensitivity to different therapeutic drugs in parallel to their clinical care. Our results showed that the therapeutic effects observed by the organoid models were consistent to the responses of these patients to their treatments. Our study suggests that PDO can recapitulate patient responses in the clinic with high potential of implementation in personalized medicine of malignant brain tumors.

## Introduction

Malignant brain tumor can be classified into two categories: the primary brain tumors such as gliomas that stem from abnormal proliferation of cells within the brain tissues, and the secondary tumors resulting from distant metastasis from the primary tumors in other organs. Among them, glioblastoma multiform (GBM) is the most common malignant primary brain tumor in adults with a poor prognosis of 5-year survival rate less than 5% following the standard therapy. In the past 17 years, the standard therapy of GBM has been surgery followed by radiation and the chemotherapeutic drug temozolomide (TMZ)^1,2^. However, patient response to the standard therapeutic protocol has been disappointing. A major factor contributing to the poor response rate is the prominent intrinsic heterogeneity of brain tumor for which the one-size-fits-all treatment strategy has fallen short despite extensive efforts^3^, calling for the need of personalized therapy.

Brain metastases are the most common intracranial tumors in adults, accounting for more than half of all brain tumors. Metastasis to the brain is an end-stage condition, a serious and debilitating complication of cancer that causes significant morbidity and mortality. Overall, 15–30% of cancer patients are diagnosed with brain metastases^4^, and consistently autopsy reveals that up to 20–40% of cancer patients develop metastatic brain tumors^5^. However, similar to GBM, metastatic tumors in the brain remain an overarching clinical challenge, and developing effective personalized therapies for BM treatment is a major unmet medical need.

A strategy to overcome this clinical challenge is to develop a “para-clinical” platform in which the patient-derived tumors can be directly tested by therapeutic drugs to empirically assess the potential responses^6^. For brain tumors, several models of glioma growth characteristics have been developed and used to investigate treatment-related cell-intrinsic pathways and mechanisms^7–10^. A major limitation of some these models resides in the incapability of the conventional GBM cell lines to recapitulate the heterogeneity of glioma stem cells (GSCs), cell–cell interactions, and treatment response. It is generally accepted that the model of patient-derived xenografts (PDXs), in which the primary tumor tissue dissected from the patient’s brain lesion is transplanted into the murine brain, more accurately mimics the tumor heterogeneity than did the other models^11–13^. However, the long latency, deviation of original oncogenes, and epigenetic alterations limit the clinical applications^14^. The recent development of PDO provides a promising preclinical model of disease^15^. It has been demonstrated that the PDOs of GBM maintain key histological, cellular, genomic, and transcriptomic features of glioblastomas, and can be deployed in a timely manner to assess patient-specific treatment responses^16^. In this study, a proof-of-concept in vitro organoid-based platform was developed and utilized to guide treatment decisions in patients with brain tumors.

## Results

**Figure 1:**
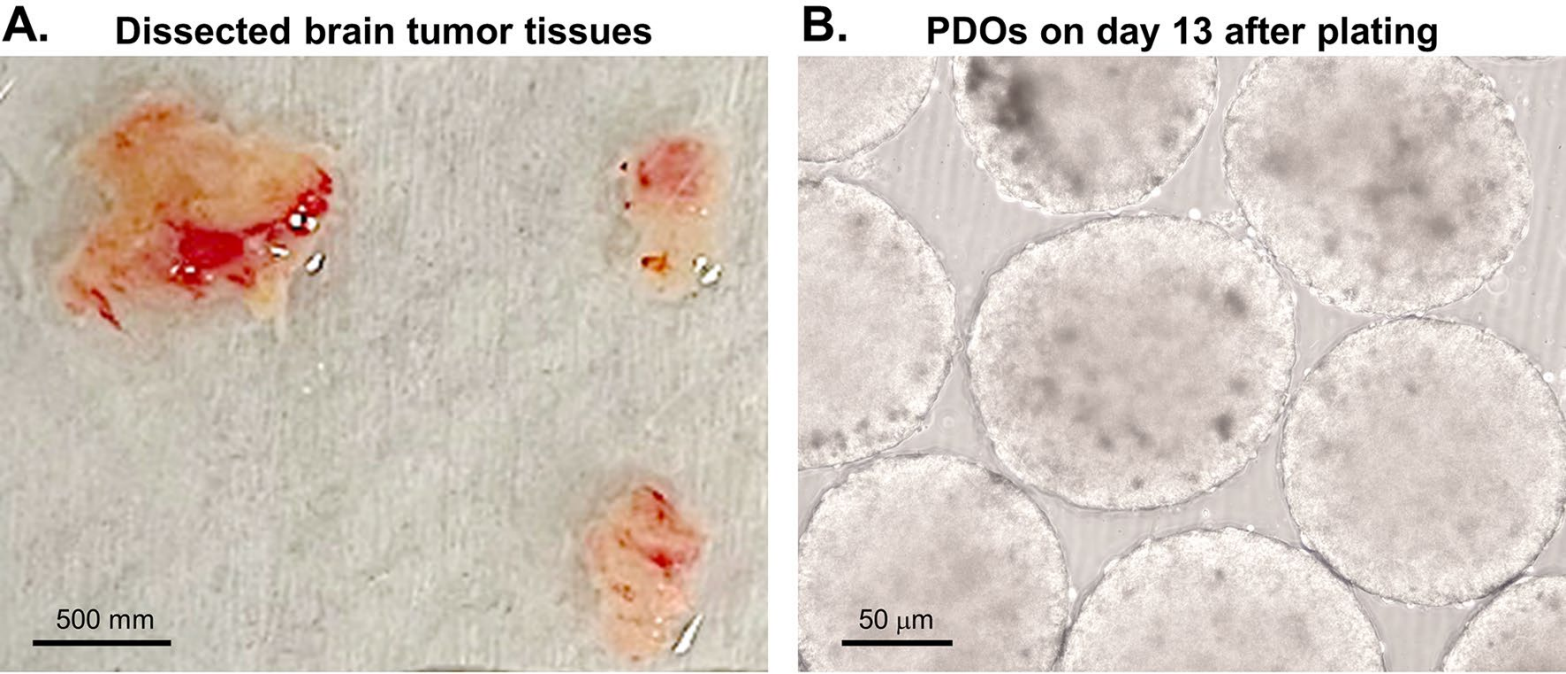
Generation of brain tumor organoids. (**A**) bright field image of dissected human brain tumor tissues. Scale bar, 500 mm. (**B**) Bright field image of representative organoids cultured for day 13 after receiving the specimen. Scale bar, 50 μm.

The 26 tumors included nine gliomas, one malignant meningioma, one primary lymphoma, and 15 metastatic brain tumors. The primary tumor sites of the brain metastases included five from the lungs, three from the breasts, two from the ovaries, two from the colon, one from the testis, one of melanoma, and one of chondrosarcoma (Table 1). The tissues were minced and plated in ultra-low attachment culture plates in the PDO media on an orbital shaker set in a cell culture incubator. Within 2 weeks, floating spherical organoids with growing sizes were visible (Fig. 1). The successful rate of PDO establishment was 50% (13/26). Compared to the matched source tumor tissues, the organoids showed similar histopathological features based on hematoxylin–eosin (H&E) staining (Suppl. Fig. 1). In this study in our institution, the dissected tumor tissues were transferred to the laboratory immediately. It normally took about 2 weeks for organoid establishment, afterwards the drug tests were completed in a week. Meanwhile, the patients received radiation therapy at the beginning of the third week after surgery. The therapy will last about 2 weeks (normally 5 doses each week). Thus, by the time the organoid tests were completed, the patients were ready to be treated by the identified treatments. This timeline is very feasible to the current clinical practice. Consistently, our finding agreed with the report by Jacob et al. that the interval time between surgical resection and laboratory processing for culturing was critical for the establishment of PDO^16^.

**Table 1:** Patient list for organoid culture in 2020–2021.

Number	Diagnosis
001	Oligodendroglioma (OA) Grade III
002	Oligodendroglioma (OA) Grade II
003	Colon CA metastasis
004	Testis CA metastasis
005	Glioblastoma (GBM) Grade IV
006	Primary CNS lymphoma
007	Anaplastic Astrocytoma Grade III
008	Anaplastic Astrocytoma Grade III
009	Lung CA metastasis
010	Breast CA metastasis
011	Breast CA metastasis
012	Lung CA metastasis
013	Lung CA metastasis
014	Ovarian CA metastasis
015	Glioblastoma (GBM) Grade IV
016	Mesenchymal chondrosarcoma metastasis
017	Glioblastoma (GBM) Grade IV
018	Ovarian CA metastasis
019	Anaplastic Astrocytoma Grade III
020	Lung CA metastasis
021	Breast CA metastasis
022	Malignant meningioma Grade III
023	Lung CA metastasis
024	Diffuse astrocytoma Grade II
025	Melanoma metastasis
026	Colon CA metastasis

**Figure 2:**
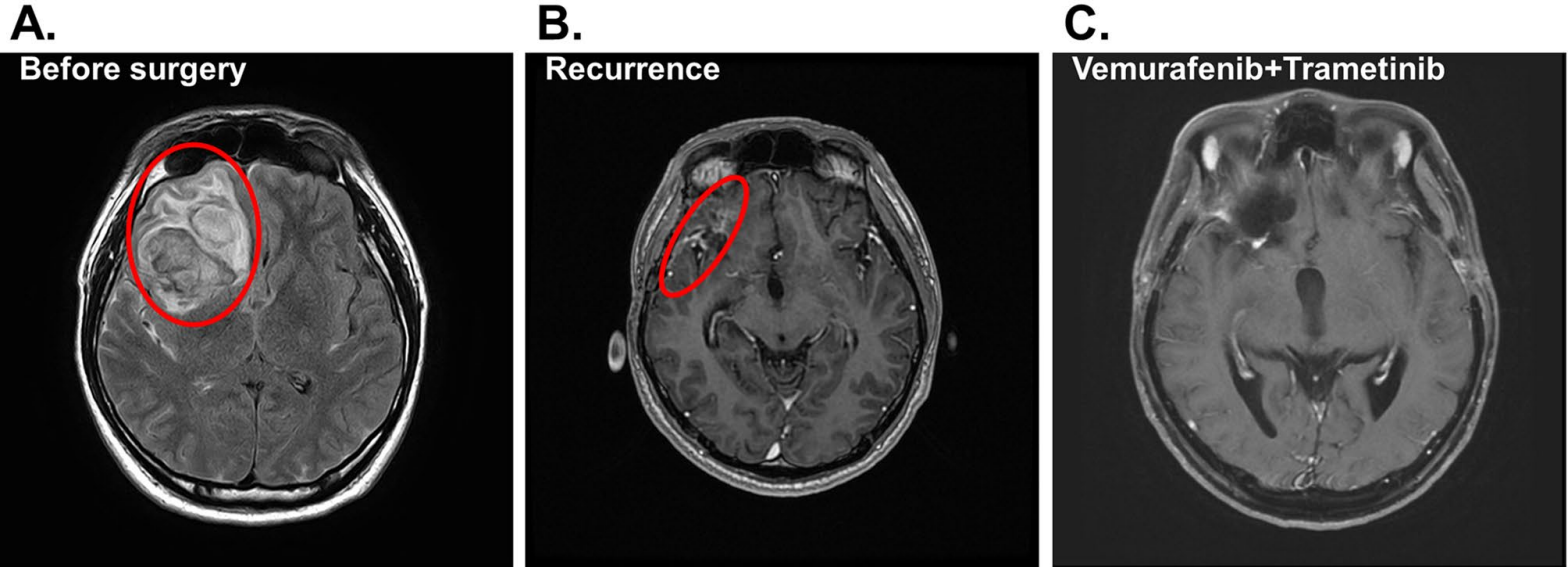
Radiological response of the brain lesion of patient #1 to BRAF inhibition. (**A**) Axial MRI before surgery with the tumor area circled. (**B**) The follow-up MRI image after surgery. (**C**) The follow-up axial image after combined treatment by vemurafenib and trametinib. Patient shows complete response.

**Figure 3:**
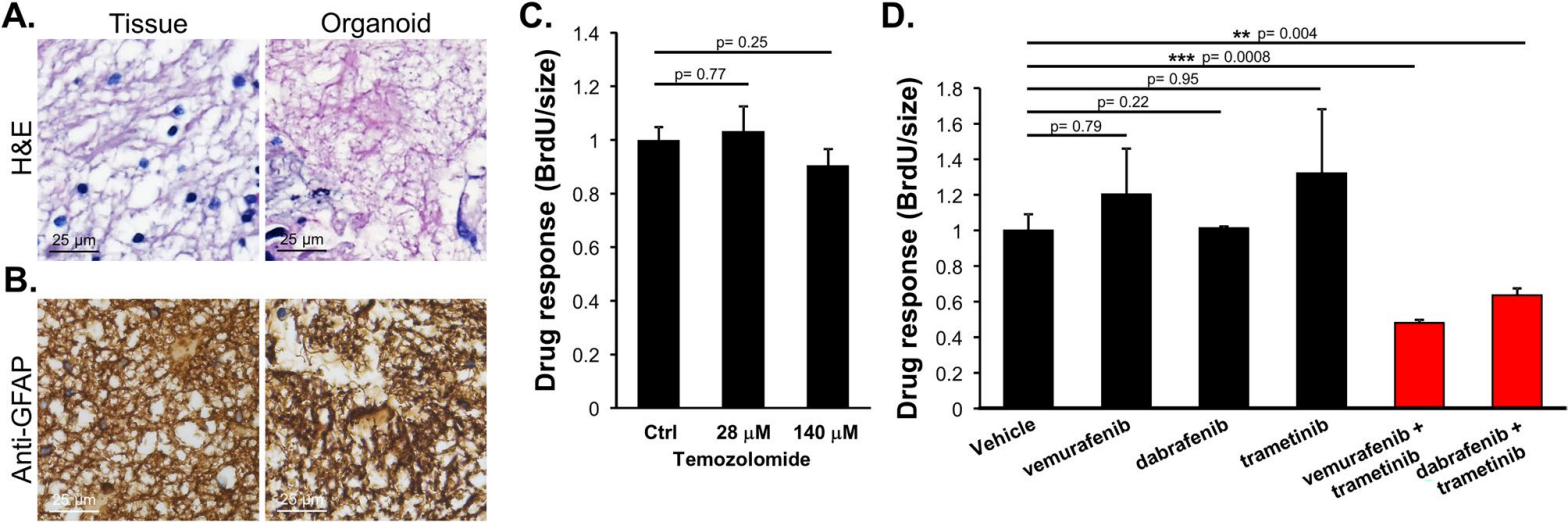
Tissue histology and drug response in the PDOs of patient #1. (**A**) H&E staining of the source tumor tissue and organoid tissue sections. Scale bar, 25 μm. (**B**) The sections were stained by immunohistochemistry for glial fibrillary acidic protein (anti-GFAP). Scale bar, 25 μm. (**C**) The PDOs were treated with temozolomide at the indicated doses converted from the clinical range. The response to the treatment was determined by BrdU incorporation assay measuring DNA replication and normalized by the dimensions of the organoids (in diameter). (**D**) PDOs were treated with vemurafenib (490 μM), dabrafenib (144 μM), trametinib (812 nM), or the indicated combinations. The response to the treatment was determined by BrdU incorporation and normalized by the dimensions of the organoids (in diameter).

Seven of the patients with metastatic cancer to the brain chose to receive standard targeted therapies corresponding to the primary cancer types and therefore their PDOs were not further pursued. Three of the remaining six patients unfortunately expired by different causes before assessing the PDOs. The remaining three PDOs were derived from patients of GBM (patient #1), metastatic brain tumor (patient #2) and low grade glioma (patient #3) and tested further with therapeutic drugs selected by the attending physicians to identify the candidate drugs for favorable responsiveness. Patient #1 was a 29-year-old male presented with a 2-month history of on-and-off headache. Subsequent investigations including MRI (magnetic resonance imaging) of the brain showed an extensive, heterogeneous infiltrative mass lesion in the right frontal temporal lobe, extending to the right parietal lobe with involvement of the cortical, subcortical and deep periventricular and subependymal regions (Fig. 2A). Craniotomy with subtotal resection was performed. Formal pathology assessment reported the diagnosis of GBM with the wild-type *isocitrate dehydrogenase IDH* gene. The intra-operatively resected tumor tissues during the first surgery were collected and immediately transferred to the laboratory for PDO culturing. The resulted PDOs and the source tumor tissues were assessed with H&E staining (Fig. 3A) and immunohistochemical (IHC) staining (Fig. 3B), showing consistent histopathological features of the two tissues.

Patient #1 then received radiotherapy (60 Gy) plus concomitant temozolomide (TMZ) over a period of six months in accordance to the National Comprehensive Cancer Network (NCCN) protocol. A follow-up MRI showed presence of residual tumor in the right frontal surgical bed (Fig. 2B). Genomic analyses of candidate genes of the tumor tissue DNA revealed the BRAF^V600E^ mutation. Thus, the three FDA-approved anti-cancer drugs targeting the BRAF mutations, vemurafenib (Zelboraf), dabrafenib (Tafinlar) and trametinib (Mekinist)^17^, were tested on the PDOs derived from patient #1 as single agents or in combinations. The results predicted that the combined treatment by vemurafenib and trametinib was the most effective (Fig. 3C,D). Based on the results, patient #1 was treated with the combination therapy of vemurafenib and trametinib. Consistent with the observation in the PDO testing, patient #1 responded to the combination therapy and the tumor disappeared as assessed by MRI (Fig. 2C). Up to now there has been no sign of recurrence for more than 13 months. The results illustrate the strong dependence of this tumor on the BRAF^V600E^ alternation. Identification of this dependency through the PDO platform confirming an actionable target for personalized treatment has expedited the process of decision-making.

**Figure 4:**
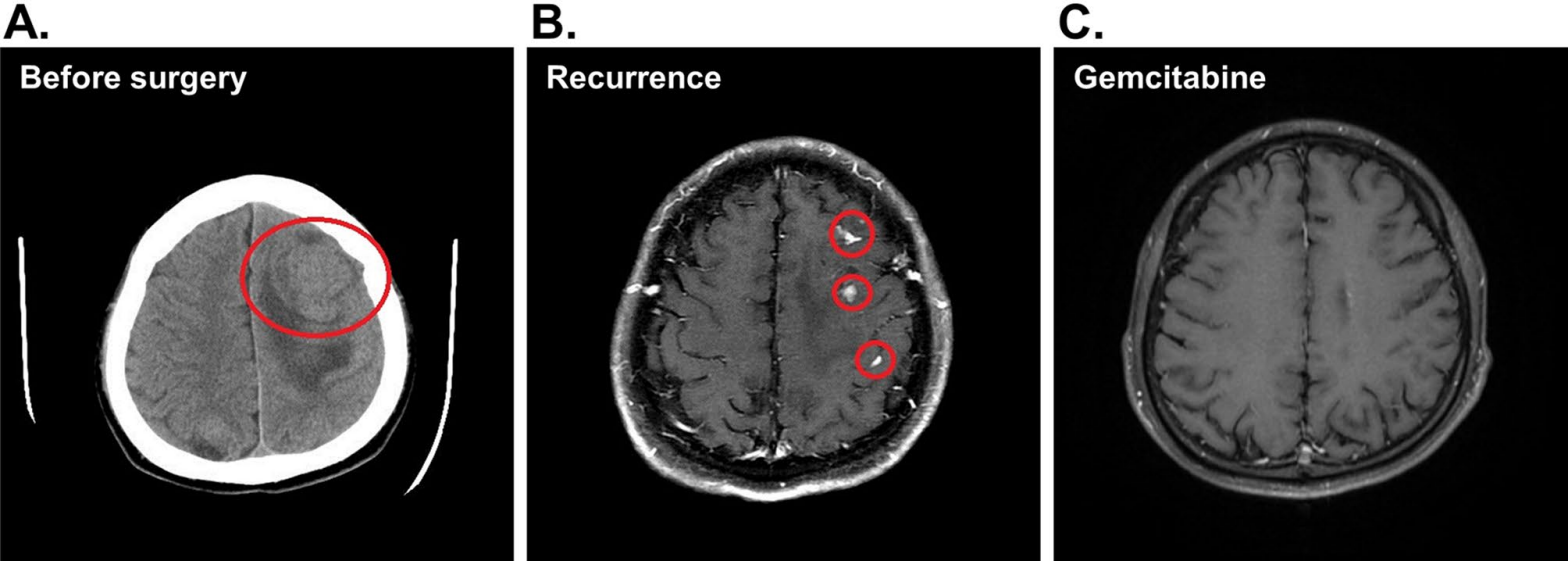
Radiological response of the brain lesion of patient #2 to gemcitabine. (**A**) A computed tomography (CT) scanning before surgery; the tumor areas are circled. (**B**) Residual lesion revealed by MRI of patient’s brain in a follow-up after surgery. (**C**) Follow-up axial MRI of the brain. The patient shows complete response after gemcitabine treatment.

**Figure 5:**
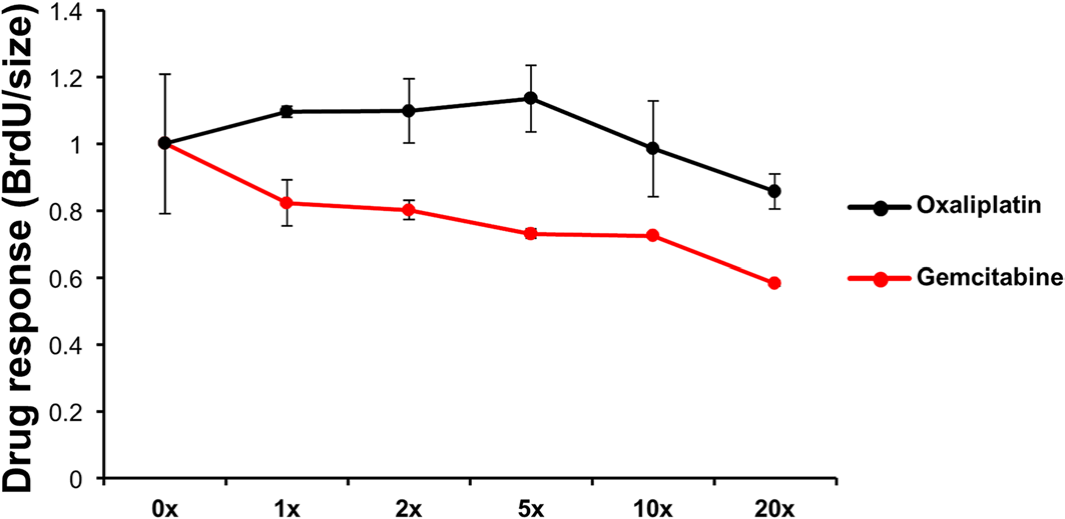
Drug response in the organoids derived from patient #2. Gemcitabine and oxaliplatin as indicated were applied to test the drug efficacy. The concentration ladders were based on the dose converted from clinical application (gemcitabine, 483 μM; oxaliplatin, 30 μM). The response to the treatment was determined by BrdU incorporation assay measuring DNA replication and normalized by the dimensions of the organoids (in diameter). Each data point was derived from two independent PDOs.

Patient #2 was a 24-year-old male diagnosed with right testicular carcinoma and lung metastasis and had been regularly receiving chemotherapy for 3 years. He suffered progressive right limbs weakness for one month before admission and the subsequent investigations by CT (computed tomography) scanning of the brain showed an extensive, big tumor on the left parietal lobe with mass effect (Fig. 4A). Craniotomy was arranged due to symptomatic right hemiparesis and the tumor was completely removed which resolved the hemiparesis. However, a follow-up MRI check showed the presence of recurrent tumor in the bilateral hemispherical brain (Fig. 4B). The intra-operatively resected tumor tissue was processed for PDOs and tested with two chemotherapy drugs gemcitabine (of Sandoz) and oxaliplatin (Orectalip). Organoid growth was assessed by BrdU incorporation assays for proliferative activity which showed that gemcitabine was more effective than oxaliplatin in growth inhibition (Fig. 5). The patient was then treated with adjustment radiotherapy (30 Gy) plus follow-up treatments by gemcitabine. Indeed, the treatment caused complete tumor regression and has no sign of recurrence for more than 15 months. (Fig. 4C).

**Figure 6:**
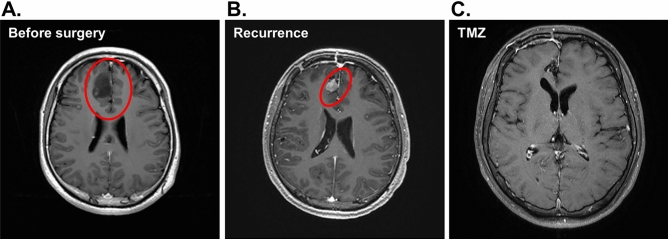
Radiological response of the brain lesion of patient #3 to temozolomide. (**A**) Axial MRI before surgery with the tumor area circled. (**B**) The follow-up MRI imaging after surgery. (**C**) Follow-up axial imaging after treatment by Temozolomide (TMZ). The patient showed complete response.

**Figure 7:**
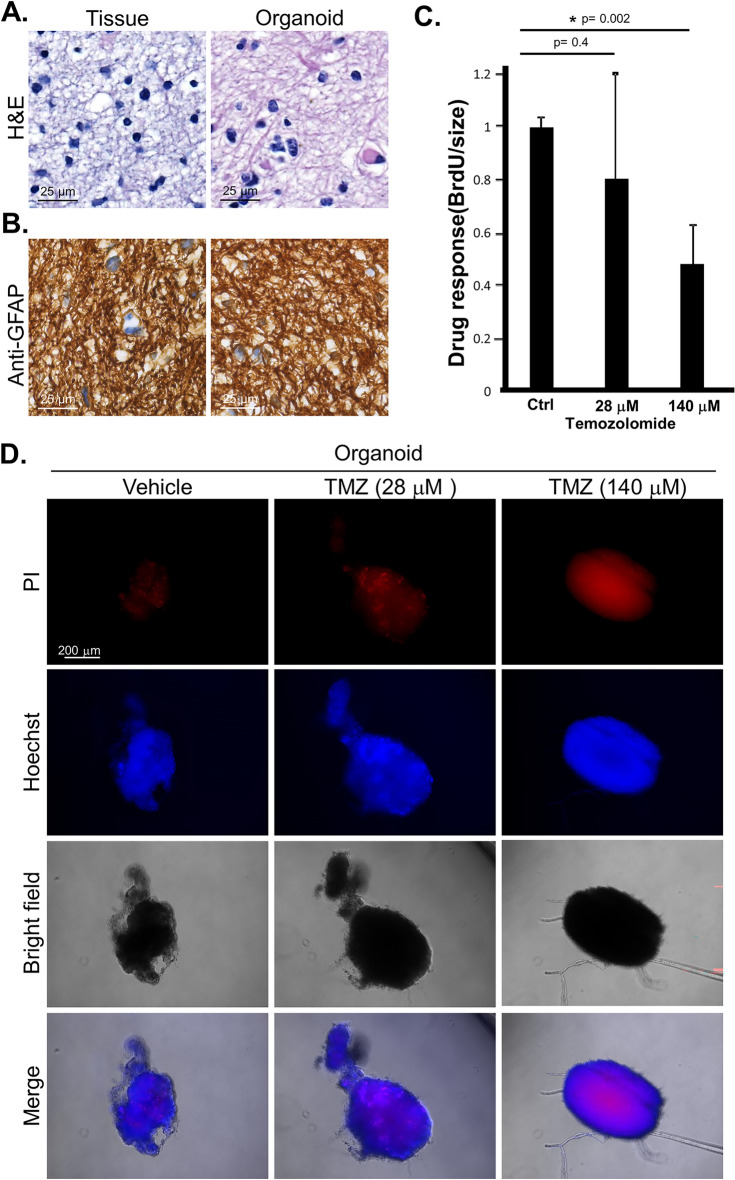
Histology of the tissue section and drug response in patient-derived organoid of patient #3. (**A**) H&E staining of the source tumor tissue and organoid tissue sections. Scale bar, 25 μm. (**B**) The sections were stained by immunohistochemistry for glial fibrillary acidic protein (anti-GFAP). Scale bar, 25 μm. (**C**) The PDOs were treated with temozolomide at the indicated doses converted from the clinical range. The response to the treatment was determined by BrdU incorporation assay measuring DNA replication and normalized by sizes of the organoid (in diameter). (**D**) Organoids were treated with temozolomide (TMZ) (28 μM and 140 μM) for 48 h. The viability of the cancer cells in the organoid was assessed by staining with Hoechst 33342 and propidium iodide (PI). Scale bar, 200 μm.

Patient #3 was a 26-year-old male presented with a 2-month history of on-and-off headache and personality changes. Subsequent investigations by MRI showed a mass at the right frontal lobe of the brain (Fig. 6A). Craniotomy with total resection was performed. Formal pathology assessment reported the diagnosis of low grade glioma with mutant *IDH*. The intra-operatively resected tumor tissues were collected and immediately transferred to the laboratory for PDO culturing. The resulted PDOs and the source tumor tissues were assessed with H&E (Fig. 7A) and IHC (Fig. 7B) staining which revealed consistent histopathological features of the two tissues. Subsequent recurrence of the tumor was detected in the right frontal surgical bed by a follow-up MRI (Fig. 6B). To assess the potential responsiveness of patient #3, the corresponding PDOs were treated with temozolomide as single agent. The proliferation activity of the PDOs treated with temozolomide was assessed by BrdU incorporation assay which showed that temozolomide significantly suppressed PDO growth (Fig. 7C). Positive staining by propidium iodide confirmed the enhanced death of the PODs by temozolomide treatment, suggesting temozolomide an effective drug of the tumor. Propidium iodide entering of the organoids was verified by Hoechst 33,342 staining in the presence and absence of propidium iodide (Fig. 7D). Consistently, patient #3 treated with temozolomide responded to the treatment with complete radiological remission as shown by MRI (Fig. 6C). To date there has been no sign of recurrence for more than 10 months.

## Discussion

Here we report the development of a PDO platform for primary and metastatic brain tumors of different original organs. We further demonstrate the promising application of the organoids in a para-clinical manner to identify the adjuvant therapeutic agents to which the recurrent patients were most likely to respond with favorable outcome.

Genomic assessment has been a principal strategy for tailoring personalized therapies into clinical practice. However, it is estimated that currently only 7% of cancer patients benefit from the genome-guided therapies^18^. The major reason for this limitation is the frequent disagreement between the prediction and real situation of clinical response. Our results suggest the superior value of combing genomic approach with the verification in the corresponding PDO before applying to the patients to attain the best achievable clinical outcomes compared to genome-guided strategies. Reasonably, using PDO to test treatment efficacy could predict human tumor responsiveness or inform additional research to delineate underpinnings of drug sensitivity. Drug screening with the PDO models takes advantage in the recapitulation of in vivo tumor biology and the increasing efficiency of organoid technology.

Many groups have also described successful biobanking of cancer organoids that can be reanimated from frozen stocks, thus allowing for the development of PDO-matched clinical databases^16,19–24^. Numerous studies have reported PDO-based drug-screening protocols, with preliminary results indicating that organoid drug responsiveness may reflect patient response^16,20,24–29^. It can be expected that the organoid platforms will be further developed to be more robust in identifying patient-specific therapeutic targets with greater clinical relevance than other models^30^.

In GBM, Jacobs and colleagues found that organoid responsiveness to gefitinib for tumors with EGFR alterations, trametinib for NF-1-mutated tumors, everolimus for PI3K-mutated tumors, and EGFRvIII-targeted CAR-T cells could be generally predictive for the response of the parent tumor^16^. Numerous case studies using organoids derived from patients, including GBM^30^, treatment-refractory peritoneal colorectal cancer^31^, and liver cancer^32^, to guide personalized therapies with success have also been reported, which also provide novel insights regarding disease mechanisms. In addition to these examples, PDO-based drug-screening protocols have been described in pancreatic, gastrointestinal, lung, prostate, ovarian, and bladder cancers^16,20,24,25,27–29,31,33–35^.

In spite of the progresses, fully translating the organoid model systems in clinical settings to direct patient care will require the overcoming of several technical hurdles, including the speed of organoid culture development, success rates of organoid establishment, cost effectiveness, throughput, and reproducibility. In current clinical practice, the time between a diagnosis of metastatic cancers via imaging and/or biopsy and the start of treatment, whether via chemotherapy, radiation, or initial resection surgery, falls optimistically in the range of 12–14 days in the United States. However, in cases of aggressive and fast-growing cancers, the diagnostic interval can be delayed for days that can lead to drastically different patient prognoses, highlighting the need for better diagnostic tools that can be implemented quickly. Thus, acceleration of the PDO development and testing timeline is necessary for these personalized medicine tools to be employed clinically.

For primary brain tumors, GBM or the low-grade glioma, a major hurdle of patient care is the limited options for drug treatments. Currently, only two drugs, temozolomide and bevacizumab (Avastin), have been approved by FDA with the indication in GBM. Implementing the para-clinical PDO-based system with the potential of high-throughput screening is expected to expand the indication of more FDA-approved chemotherapeutic and targeted drugs for brain tumor therapy.

## Conclusion

We have conducted a prospective clinical study demonstrating the robust potential of PDO system derived from primary and metastatic brain tumors to identify the regimen the tumor is likely to respond to and therefore enhance the patient survival. Our data suggest that PDO can recapitulate patient responses in the clinic, and have the potential to be implemented in personalized medicine programs.

## Methods

### Patients

During December 2020 to October 2021, 26 malignant brain tumor tissues (Table 1) were isolated by craniotomy resection and processed for PDO cultures following the approved IRB protocol of the China Medical University Hospital, Taichung City, Taiwan (CMUH109-REC3-173). All experiments were performed in accordance with the guidelines and regulations of CMUH. Informed consent was obtained from all participants or their legal guardians.

### Tumor processing and PDO culture

The tumor tissues were processed for organoid culturing according to the protocol published by Jacob et al.^16^. Briefly, the tumor pieces were transferred to ultra-low cell culture plates (Corning) containing 50% DMEM:F12 (Cat. #11320033; GIBCO, Waltham, USA), 50% Neurobasal (Cat. #21103049; GIBCO, Waltham, USA), 1 × GlutaMax (Cat. #35050061; GIBCO, Waltham, USA), 1 × NEAAs (Cat. #41500018; GIBCO, Waltham, USA), 1 × PenStrep supplement (Cat. #15140112; GIBCO, Waltham, USA), 1 × N2 supplement (Cat. #17502048; GIBCO, Waltham, USA), 1 × B27 without vitamin A supplement (Cat. #12587010; GIBCO, Waltham, USA), 1 × 2-mercaptoethanol supplement (Cat. #21985023; GIBCO, Waltham, USA), in the presence of 2.5 mg/ml human insulin (Cat. I9278; Sigma-Aldrich, St. Louis, USA). The plates were placed on an orbital shaker rotating at 120 rpm set up in a sterile incubator with 5% CO_2_, 90% humidity at 37 °C. Most of the medium (~ 75%) was changed every 48 h with fresh medium with minimal disturbance of the organoids. The criteria for successful establishment of PDO from a given patient’s tumor is that the micro-dissected tumor pieces survived for 2 weeks, being able to develop a spherical morphology, and continuously propagating in culture. The PDOs are propagated by dissecting to pieces of 50–100 μm in diameter using fine dissection scissors to prevent necrosis within the organoid center due to limited nutrient and oxygen diffusion. It took around 2 weeks for organoid culturing after surgical resection of the tumor tissues before testing the selected drugs. It would take another 1–2 weeks to decide the drugs to be applied for adjuvant treatment. Three patients (patient #1/CCCG015, patient #2/CCCG004, patient #3/CCCG024) and the corresponding PDOs were further pursued para-clinically. The tumor tissue of patient #1 was analyzed by the FoundationOne® CDx genomic assay (Foundation Medicine, Inc.)

### Bromodeoxyuridine (BrdU) incorporation assay for organoid growth

The assay was conducted by following the manufacturer’s protocol (Roche). Briefly, organoids were seeded in 96-well microplate in appropriate sizes, ~ 50 μm in diameter by micropipette. BrdU was added to the culture and incubated for 24 h in a sterile incubator with 5% CO_2_, 90% humidity at 37 °C. The microplate was centrifuged at 300 × *g* for 10 min and the medium was removed. The residual medium was removed by placing the microplate in an oven at 60 °C for about 1 h following the manufacturer’s instruction (Roche, Cat. No. 11-647-229-001). The heating is necessary to dry the residual medium is dried out, which is important to retain the organoid tissues in the subsequent processing steps. Organoids were fixed in FixDenat solution for 30 min at room temperature, followed by incubating with the anti-BrdU-POD solution for 1.5 h at room temperature. After washing with PBS for three times, the substrate solution was added and incubated for 30 min until appropriate color developed. The reaction was terminated by 1 M H_2_SO_4_ and the absorbance at 450 nm was measured by a spectrometer. For the quantification of cell growth, the BrdU data were normalized by its initial organoid diameter.

### Hematoxylin and eosin stain (H&E)

Formalin-fixed paraffin-embedded tissue sections (5 μm in thickness) were heated at 65 °C for 1 h. The tissue sections were deparaffinized by xylene, followed by hydration through a concentration gradient of alcohol for 3 min. The hydrated slides were washed in water twice, followed by staining with hematoxylin for 3 min. The slides were then washed in running water for 10 min, followed by eosin staining for 3 min. Followed by dehydration through serial incubations in ethanol of 75% (3 min), 95% (1 min), 95% (3 min), 100% (1 min), and 100% (3 min), the slides were washed twice in xylene (10 min each), then mounted for examination under microscope.

### Immunohistochemistry (IHC)

Organoids were fixed in 10% neutral buffered formalin and then embedded in paraffin. The blocks were cut into 5-μm thick coronal sections. Tissue sections were first placed in BOND-III automated IHC (Leica Biosystems) and incubated with primary antibodies against glial fibrillary acidic protein (GFAP) (GFAP-GA5-L-CE, Leica Biosystems), followed by biotinylated secondary antibodies (Biotinlated goat anti-mouse IgG) and incubation with avidin-biotinylated complex. DAB substrate was used as the detection reagent (EW-93951-85, Vector).

### Detection of organoid death by propidium iodide and Hoechst 33342 staining

PDOs were washed by 1 × PBS, and stained by Hoechst33342 (Invitrogen) and PI (propidium iodide, Invitrogen) for 1 h. Fluorescence was captured by inverted fluorescence microscopy under a magnification of 400×.

## Supplementary Information

Supplementary Legends.

Supplementary Figure 1.

## Data Availability

The datasets generated during the current study are available from the corresponding authors on reasonable request.
