# Supplementary material for: Patient-derived tumor organoids as a platform of precision treatment for malignant brain tumors
Source: Sci Rep. 2022 Sep 30;12:16399. doi: 10.1038/s41598-022-20487-y (PMC9525286; doi:10.1038/s41598-022-20487-y)
Supplement: Supplementary file 1 — Supplementary Legends. [file 41598_2022_20487_MOESM1_ESM.docx]

**Supplementary Figure 1. PDOs maintained similar histopathological features of the paired source tumor tissues.** Representative H&E staining images of brain tumor organoids and the corresponding source brain tumor tissues. CCCG015, patient #1; CCCG024, patient #3. Scale bar, 100 μm.
