# Supplementary figures and images for: Patient-derived tumor organoids as a platform of precision treatment for malignant brain tumors
Source: Sci Rep. 2022 Sep 30;12:16399. doi: 10.1038/s41598-022-20487-y (PMC9525286; doi:10.1038/s41598-022-20487-y)

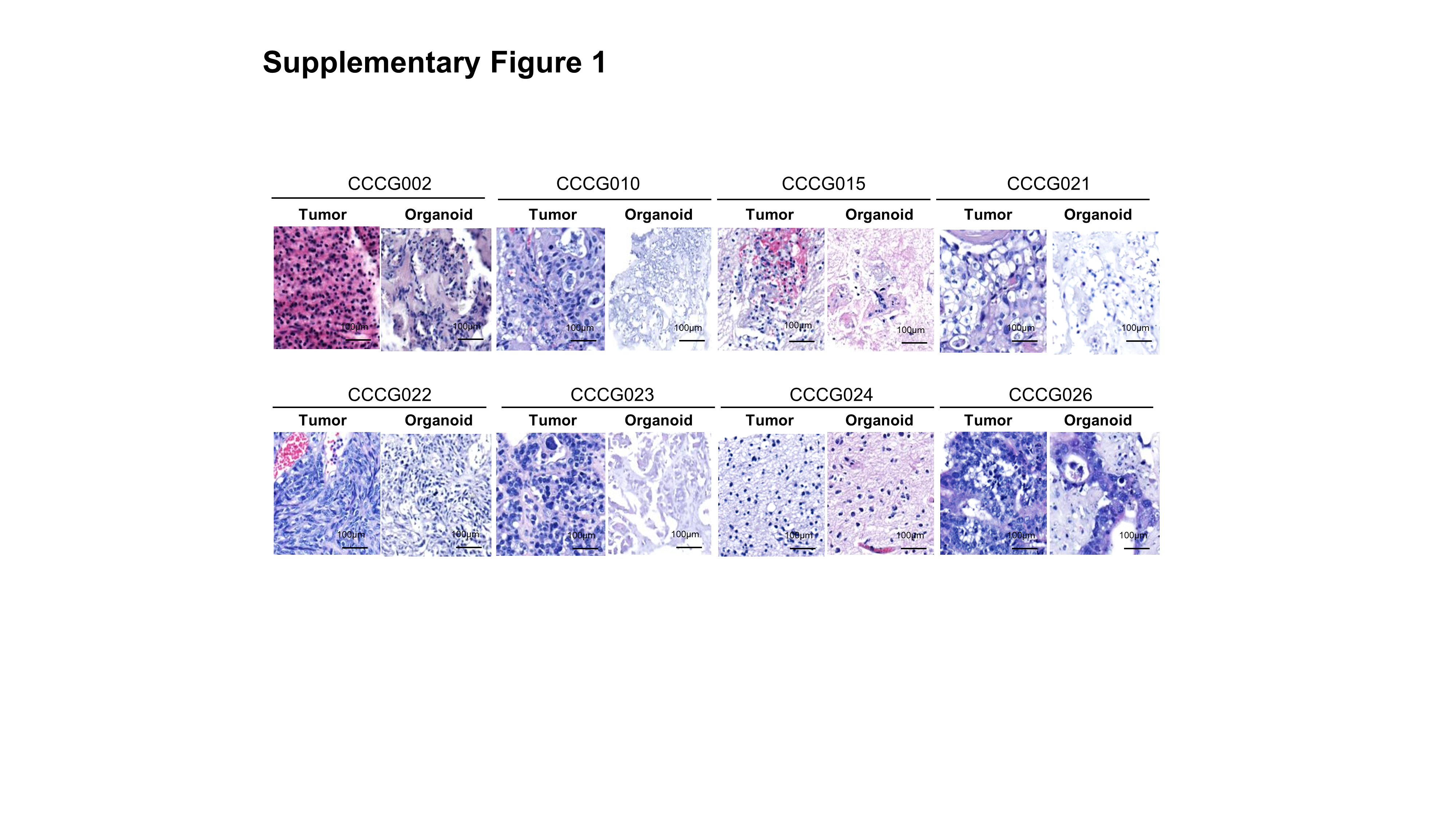

Supplement: Supplementary file 2 — Supplementary Figure 1. [file 41598_2022_20487_MOESM2_ESM.tif]
